# Iron–sulfur clusters have no right angles

**DOI:** 10.1107/S205979831801519X

**Published:** 2019-01-04

**Authors:** Nigel W. Moriarty, Paul D. Adams

**Affiliations:** a Molecular Biosciences and Integrated Bioimaging, Lawrence Berkeley National Laboratory, Berkeley, CA 94720, USA; b Department of Bioengineering, University of California, Berkeley, CA 94720, USA

**Keywords:** macromolecular refinement, iron–sulfur clusters, restraints

## Abstract

Accurate geometric restraints are vital in the automation of macromolecular crystallographic structure refinement. A set of restraints for the Fe_4_S_4_ cubane-type cluster was created using the Cambridge Structural Database (CSD) and high-resolution structures from the Protein Data Bank. Geometries from each source were compared and pairs of refinements were performed to validate these new restraints. In addition to the restraints internal to the cluster, the CSD was mined to generate bond and angle restraints to be applied to the most common linking motif for Fe_4_S_4_: coordination of the four Fe atoms to the side-chain sulfurs of four cysteine residues. Furthermore, computational tools were developed to assist researchers when refining Fe_4_S_4_-containing proteins.

## Abstract 2

A set of restraints for an iron–sulfur cluster based on small-molecule structures was generated and tested in structure refinement. Additionally, the small-molecule structures also provided bond and angle restraints for linking the cluster to the coordinating cysteine residues.

## 1. Introduction   

Using accurate geometric restraints is essential in macromolecular crystallography in order to arrive at chemically meaningful atomic models. The experimental data, even when available at very high resolution, are typically unable to unambiguously define the exact conformation, and therefore prior chemical knowledge is included in the form of geometric restraints. Relying on quantum calculations to help to define these restraints can be very productive (Moriarty *et al.*, 2009 ▸), but for metal clusters the challenge usually exceeds the available resources because of the high basis-set levels that are required for accurate calculations, not to mention the variability in possible geometries. Therefore, the use of high-quality experimental data, typically from small-molecule crystallo­graphy, to generate restraints and subsequent validation using a large number of refinements is a common paradigm. This procedure generally makes uses of the r.m.s. deviation between the target restraints and the refined models as a metric. We have used this approach to define accurate restraints for iron–sulfur clusters.

**Figure 1:**
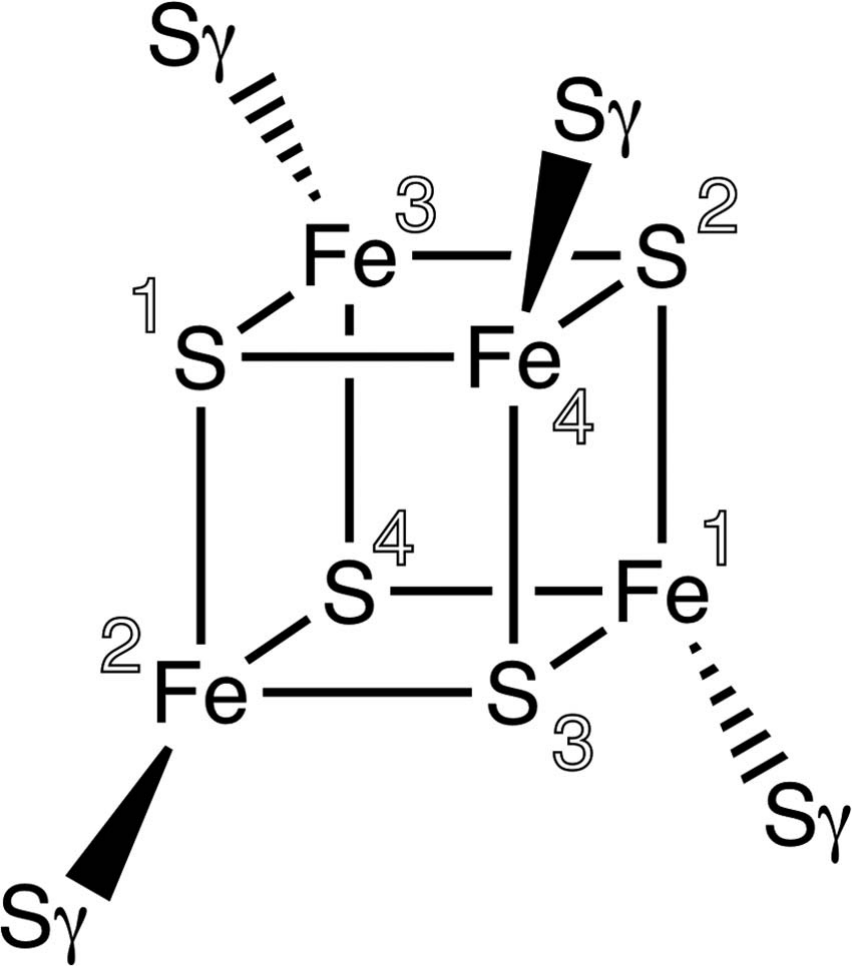
Representation of SF4 including the links to side-chain S atoms of cysteine residues. Numbers represent the atom names ranging from Fe1 to Fe4 and from S1 to S4.

Iron–sulfur clusters occur in a variety of proteins with diverse functions, including electron transfer, control of gene expression, substrate binding and redox chemistry (Bruschi & Guerlesquin, 1988 ▸; Nogi *et al.*, 2000 ▸; Cherrier *et al.*, 2014 ▸). Multiple types of iron–sulfur cluster have been observed. However, the most common is an Fe_4_S_4_ cubane-type cluster, which is typically represented chemically as a cubic structure, with the ligating sulfurs coming from the protein (see Fig. 1 ▸). The central role of the cluster in multiple biological functions makes crystallography an attractive tool for investigating the details of their mechanisms. In addition, the presence of a metal-containing cluster in a protein can also be an aid to structure solution using anomalous scattering. Clearly, the use of accurate geometric restraints for any iron–sulfur cluster is essential for obtaining high-quality atomic models for these important classes of proteins. Encountering some unusual restraints in available crystallographic libraries for iron–sulfur clusters prompted us to derive new restraints using small-molecule structures, and to test these new restraints by the re-refinement of nearly 240 iron–sulfur-containing crystal structures.

## 2. Methods   

When developing accurate experimental ligand geometries, there are two main sources of information in the field of macromolecular crystallography. One choice is small-molecule structure databases such as the Cambridge Structural Database (Groom *et al.*, 2016 ▸) or the Crystallography Open Database (Gražulis *et al.*, 2009 ▸). The other choice is the very high-resolution macromolecular structures in the Protein Data Bank (PDB; Berman *et al.*, 2000 ▸). Both have their pros and cons (Long *et al.*, 2017*a* ▸), but in this study both the CSD and the PDB were used.

**Figure 2:**
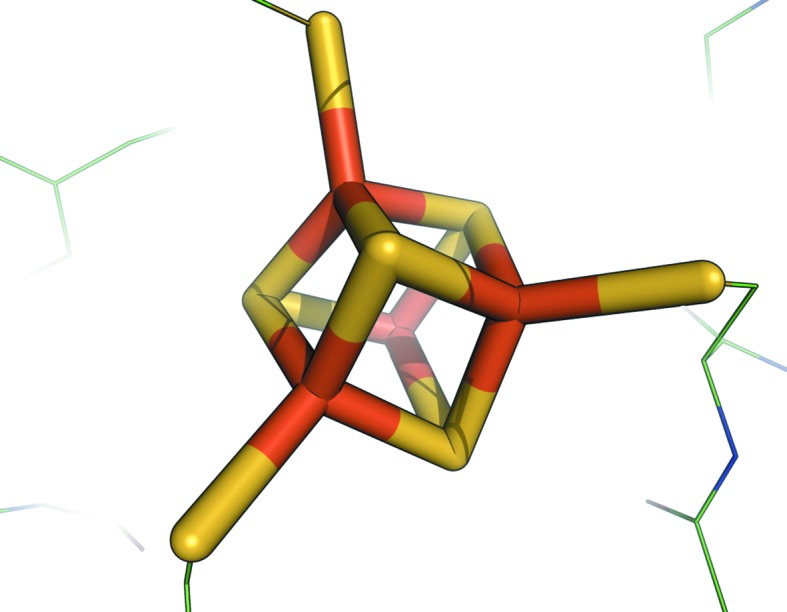
Graphical representation of the high-resolution geometry of SF4 in PDB entry 1iUA produced by *PyMOL* (DeLano, 2002 ▸).

The most prolific iron–sulfur cluster, which has the residue name SF4 and has 855 entries in the PDB as of December 2017, has the chemical formula Fe_4_S_4_, with each element forming only heterogeneous bonds (see Fig. 1 ▸). It is commonly coordinated *via* the Fe atoms to four S atoms of cysteine residues in the macromolecule. The ideal coordinates in the Chemical Components Dictionary (CCD; Westbrook *et al.*, 2015 ▸) suggest that the cluster is a perfect cube with 90° angles for both the S—Fe—S and Fe—S—Fe angles. However, high-resolution structures containing SF4 such as PDB entry 1iUA 1 (Liu *et al.*, 2002 ▸) have a distinctly noncubic geometry. Fig. 2 ▸, produced in *PyMOL* (DeLano, 2002 ▸), shows the non­cubic nature of the cluster and the commonly linked sulfurs from cysteine residues. Furthermore, it highlights the fact that the Fe atoms are typically coordinated to four S atoms, thus requiring a non­­cubic geometry.

It should be noted that the CCD has fields for two sets of Cartesian coordinates: one set is the ideal coordinates and the other is taken from the PDB structure with the best resolution, the code for which is listed elsewhere in the file. Either or both can be absent, so heuristics are required to extract Cartesian coordinates from the CCD file. The current CCD entry has ideal coordinates that are cubic and experimental coordinates from an unknown PDB entry that are approximately rhombo­hedral. The restraints in the Monomer Library v.4.51 (Vagin *et al.*, 2004 ▸) and those used in *Coot* 0.8.8 (Emsley *et al.*, 2010 ▸) are both cubic. Interestingly, the obsoleted predecessor of SF4, F4S, had approximately rhombohedral restraints, but these restraints have been removed from the Monomer Library.

Generating restraints for clusters can be challenging. At present, *AceDRG* (Long *et al.*, 2017*b* ▸) cannot generate restraints for any compound that contains metals. *Grade* (Smart *et al.*, 2011 ▸) also is unable to provide restraints for iron-containing compounds. The *electronic Ligand Builder & Optimization Workbench* (*eLBOW*; Moriarty *et al.*, 2009 ▸) can generate restraints for clusters if an accurate three-dimensional starting model is supplied.

An additional challenge is the high symmetry of the cluster. This means that the atom naming is not unique, as each of the Fe atoms is chemically identical. However, the correct application of the geometric restraints relies on correct atom naming, which can unfortunately be permuted in the model file. We observe that this is a problem in the PDB, where the atom naming for some SF4 clusters is at odds with the restraint geometries, as described in Section S1 of the supporting information. Visual detection of these discrepancies is straightforward with the restraints editor *REEL* (Moriarty *et al.*, 2017 ▸), which displays the geometric restraints (Afonine & Moriarty, 2016 ▸) rather than drawing bonds based on the relationship of an atom to other atoms in space.

To obtain an accurate geometry for SF4, the CSD was interrogated using a structure search in *Conquest* (Bruno *et al.*, 2002 ▸) with the topology of an SF4 cluster coordinated via the Fe atom to a single S atom. Note that this search excludes structures containing iron coordinated to more than one external atom or coordinated to π orbitals such as aromatic rings. Using the strictest criteria for *R* factors (≤0.05) and other search options, 24 CSD entries with 25 instances were identified and denoted as search S(0.05). The other filter options used were three-dimensional structures available and no errors. A second search is designated X(0.05) because the cluster could be linked to any atom. Using the same filters, 60 entries with 62 instances were selected. Relaxing the search criteria to X(0.1) resulted in 114 CSD entries and 123 instances. Further analysis was performed with *Mercury* (Bruno *et al.*, 2002 ▸; Macrae *et al.*, 2006 ▸, 2008 ▸). The QUEST query file from the first search, S(0.05), and the filter settings are provided in Supplementary Fig. S3 and Supplementary Table S1, respectively.

Interrogation of the high-resolution structures in the PDB was also performed using a custom-written Python script to determine the ideal geometry of SF4 as a comparison to the CSD methodology. Using high-resolution structures in the PDB that contain SF4 produces the results shown in Table 2. After filtering for structures better than 1 Å resolution that have deposited structure factors and other miscellaneous items such as having all atoms present in the cluster, there are six entries.

## 3. Results   

The three structure searches of the CSD (Groom *et al.*, 2016 ▸) for bond and angle values for the Fe_4_S_4_ cluster SF4 resulted in the values and statistics given in Table 1 ▸. The bond distance for S—Fe for the strictest search is 2.29 ± 0.02 Å, which is essentially the same as the values for the other two searches, X(0.05) and X(0.1), and for the coordinating bonds (Fe—S_AA_). The bond distance from the PDB search is 2.30 ± 0.03 Å, which is in close agreement with the CSD results. This agrees well with the results reported in Tan *et al.* (2013 ▸), which list all bond lengths for clusters ligated to sulfur as spanning these values. The value is also in good agreement with that posted by Oliver Smart to the CCP4 bulletin board in 2014: 2.298 Å.

The values for S—Fe—S angles, however, differ between the internal and external (S—Fe—S_AA_) angles: 104.2 and 114.2°, respectively. The standard deviation of the external angle is 5.8°, which is much larger than the value of 1.2° for internal angles. The inclusion of any coordinating atom does not significantly affect the bond and angle values, but increases the standard deviations. The ligand does affect both the bond and values in the study by Tan *et al.* (2013 ▸), who note that ‘non-innocent’ and strongly covalent ligands make the bond lengths shorter (as short as 2.2 Å) and that the bond angles range from 80 to 100°. This is particularly noticeable for SF4 coordinated to π orbitals of negatively charged ring structures. The PDB results (Table 2 ▸) are very similar to the results from the CSD. Restraints were generated using the CSD values for bonds and angles. Chiral restraints were also included to retain the same orientation of all SF4 clusters.

The resulting restraints taken from the CSD values were tested by performing refinements using *phenix.refine* (Afonine *et al.*, 2012 ▸) of all suitable models containing SF4 in the PDB. This includes the use of the linking restraints when the SF4 was in the presence of four cysteine amino acids. Two sets of refinements of all SF4-containing structures from the PDB solved at 3.55 Å or better that satisfied the following criteria were performed. Firstly, they had to have diffraction data deposited that were not twinned, were ≥90% complete and could be successfully converted to an MTZ file format. Secondly, they had to have starting calculated *R*_work_ and *R*_free_ values that were less than 30% and 35%, respectively, and an *R*_free_ − *R*_work_ difference of ≥1.5%, with the latter criterion serving to filter out structures that may not have the correct *R*_free_ test set deposited. Applications of these filters provided 239 PDB entries. One set of refinements was performed using the cubic restraints from the Monomer Library, while the other was performed using the rhomboid restraints based on the CSD values. The latter restraints were generated using *eLBOW* (Moriarty *et al.*, 2009 ▸) and edited to match the CSD values using *REEL*. The refined bond lengths and bond angles for each model are given in two CSV files in the supporting information.

One of the complications when refining an SF4 cluster is that the atom naming is critical to restraining the geometry, but the symmetry of the cluster makes it easy to create a starting model that is not in the local minimum specified by the restraints, *i.e.* the atom names can be misassigned. In such cases it is likely that the SF4 cluster cannot be optimized to the correct minimum and highly distorted geometries are produced. To reduce the effect of incorrect atom naming, a feature was added to *PHENIX* (Adams *et al.*, 2010 ▸) that superposes the ideal geometry of the SF4 in the correct configuration on the input model and renames the atoms appropriately. This reduces the manual intervention required for SF4 refinement, and the same approach could be applied to other small-molecule entities in the future. This procedure was performed for both the Monomer Library restraints, which specified the ideal bond length as 2.135 ± 0.020 Å, and the CSD value restraints (top section of Table 1 ▸).

An additional feature was added to the automatic linking algorithms in *PHENIX*. As discussed earlier and shown in Table 1 ▸, the S—Fe—S angle involving the coordinating cysteine sulfur is 114.2°, which differs from the internal value. Therefore, if the SF4 cluster is linked to a cysteine sulfur via an Fe atom in SF4 in a model, the values of both the linking bond and linking angle shown in Table 1 ▸ are applied to the model. Since the calculations were performed for this publication, the ideal bond values for Fe—S with the sulfur in other entities such as MET have been amended.

## 4. Discussion   

**Figure 3:**
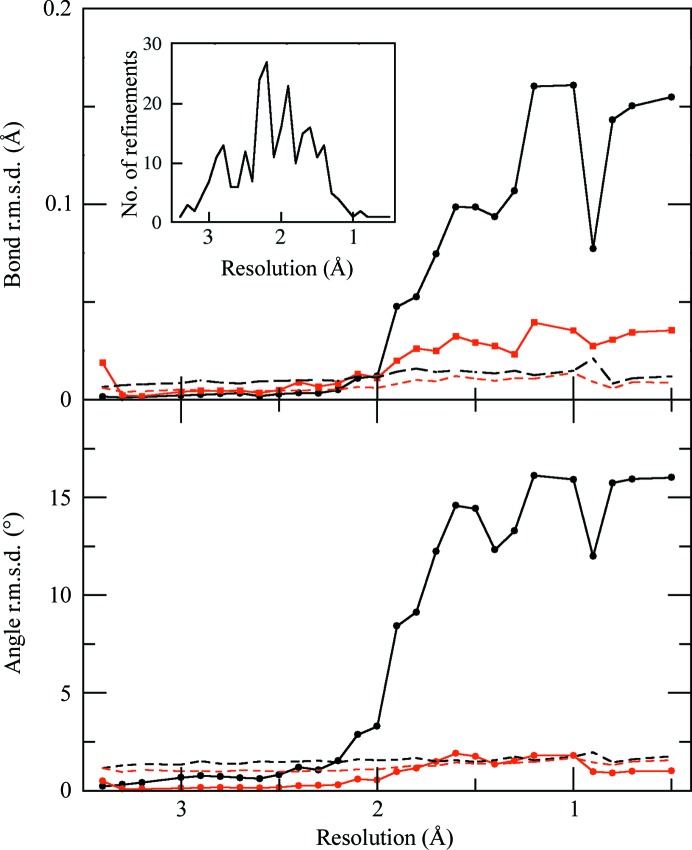
Plots of bond r.m.s.d. (top) and angle r.m.s.d. (bottom) values for the entire model (dashed lines) for both the cubic restraints (black) and rhomboid restraints (red). The r.m.s.d. values for just the SF4 iron–sulfur clusters are shown as a black line with circle markers for cubic restraints and as a red line with circle markers for rhomboid restraints. The number of refinements in each resolution bin is shown in the inset plot.

The 239 PDB entries cover the resolution range from 0.5 to 3.4 Å, with the best coverage from 1.3 to 3.0 Å. Most of the SF4 geometries were rhomboid but 23, or nearly 10%, were cubic, with an additional 15 (6.3%) of input models having incorrect atom naming. Most metrics such as *R* factors, Ramachandran, rotamer and clashscore are similar, with some noise in the limits. However, the bond and angle r.m.s.d. values show significant variations. The bond and angle r.m.s.d. values for the entire models (dashed lines) are shown in Fig. 3 ▸. The r.m.s.d. values for the entire model change very little with respect to resolution owing to the limited impact of a small number of deviations corresponding to the metal clusters, but as expected there is a small increase at high resolution because the data provide more information to define the final geometry.

Turning to the r.m.s.d. values specifically for the metal cluster, for data sets at worse than 2 Å resolution the r.m.s.d. values for the two restraint (rhomboid and cubic) sets are very similar, as the paucity of experimental data requires that the refinement algorithms rely on the geometric restraints to define the geometry of the iron–sulfur clusters. This highlights that the use of incorrect geometric restraints with low-resolution data cannot be readily detected by analysis of the deviations between the model and the target restraints. The use of truly cubic restraints with low-resolution data would most likely lead to models with cubic iron–sulfur geometries, which would be unrecognized as an error without more detailed analysis.

## 5. Conclusions   

New restraints using a rhomboid geometry have been added to the GeoStd restraints (N. W. Moriarty & P. D. Adams; https://sourceforge.net/projects/geostd/) for use in all *PHENIX* programs from v.1.13. The restraints can also be loaded into *Coot*. Both the CSD values and the PDB values were accurate for macromolecular refinement, with the former being demonstrated to provide improved geometries.

Two additional features, the automatic superposition of the correct atom naming and the addition of dynamic SF4–cysteine linking, including bonds and angles, have been added to *PHENIX*.

## Supplementary Material

Internal coordinates for SF4 using CSD restraints.. DOI: 10.1107/S205979831801519X/rr5165sup1.txt

Internal coordinates for SF4 using cube restraints.. DOI: 10.1107/S205979831801519X/rr5165sup2.txt

Use of REEL, Supplementary Figures and Supplementary Table.. DOI: 10.1107/S205979831801519X/rr5165sup3.pdf

## Figures and Tables

**Table 1:** Geometry values for SF4 from the Cambridge Structural Database using three searches denoted S(0.05) for SF4 linked to sulfur and *R* factor ≤ 0.05, X(0.05) for SF4 linked to any element and *R* factor ≤ 0.05, and X(0.1) for any link element and an *R*-factor cutoff increased to 0.1 Bond lengths are in ångströms and angles are in degrees.

	Mean	Standard deviation
S-linked, S(0.05)		
S—Fe	2.289	0.024
Fe—S—Fe	73.66	0.87
S—Fe—S	104.18	1.24
Fe—S_AA_	2.268	0.017
S—Fe—S_AA_	114.24	5.75
X-linked, X(0.05)		
S—Fe	2.285	0.027
Fe—S—Fe	73.39	1.05
S—Fe—S	104.38	1.33
S—Fe—X	114.30	4.73
X-linked, X(0.1)		
S—Fe	2.284	0.029
Fe—S—Fe	73.71	1.52
S—Fe—S	104.14	1.50
S—Fe—X	114.29	5.24

**Table 2:** Geometry values for SF4 from high-resolution structures (<1 Å) in the PDB Bonds are in ångströms and angles are in degrees.

	Mean	Standard deviation
S—Fe	2.286	0.028
Fe—S—Fe	72.98	0.62
S—Fe—S	104.69	0.89
Fe—S_AA_	2.266	0.014
S—Fe—S_AA_	113.78	6.16

## Footnotes

1 Both the PDB protein and ligand codes follow the convention outlined in Moriarty (2015 ▸).
