# Supplementary material for: Iron–sulfur clusters have no right angles
Source: Acta Crystallogr D Struct Biol. 2019 Jan 4;75(Pt 1):16–20. doi: 10.1107/S205979831801519X (PMC6333285; doi:10.1107/S205979831801519X)
Supplement: Supplementary file 3 [file d-75-00016-sup3.pdf]

STRUCTURAL BIOLOGY

Volume 74 (2018)

Supporting information for article:

Iron–sulfur clusters have no right angles

Nigel W. Moriarty and Paul D. Adams

S1. Using REEL to validate input models and restraints

One of the features of the restraints editor, REEL (N. W. Moriarty, Draizen, and Adams 2017), is loading a set of restraints and the Cartesian coordinates from a PDB file into the same view. In REEL, the bond representation is drawn based on the bond restraints list in the restraints file and not on any heuristics such as bond distance cut-offs that often lead to misleading visuals (Afonine and Moriarty 2016). Loading the restraints for SF4 distributed with the Monomer Library v5.41 (Vagin et al. 2004) and the experimental coordinates from PDB entry 1iUA results in figure S1. Note that, as discussed in the main text, the Cartesian coordinates are not in a cubic configuration.

If, however, the same procedure is repeated using the coordinates from PDB entry 2FLA, the bonds are connecting atoms on opposite sides of the cluster (see figure S2). Because refinement will move to the closest minimum, the resulting geometry of the SF4 cluster will be far from ideal.

REEL, then, gives a strict representation of the bonds used in a refinement. This feature can be used to validate the relationship between the model geometry and restraints of any entity.

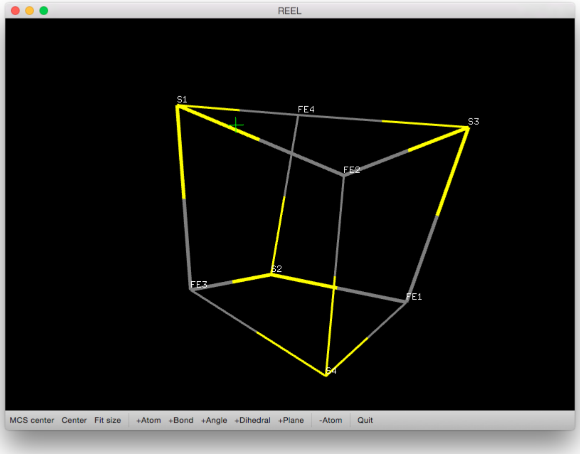

Figure S1 Loading coordinates from 1iUA into the standard Monomer Library restraints.

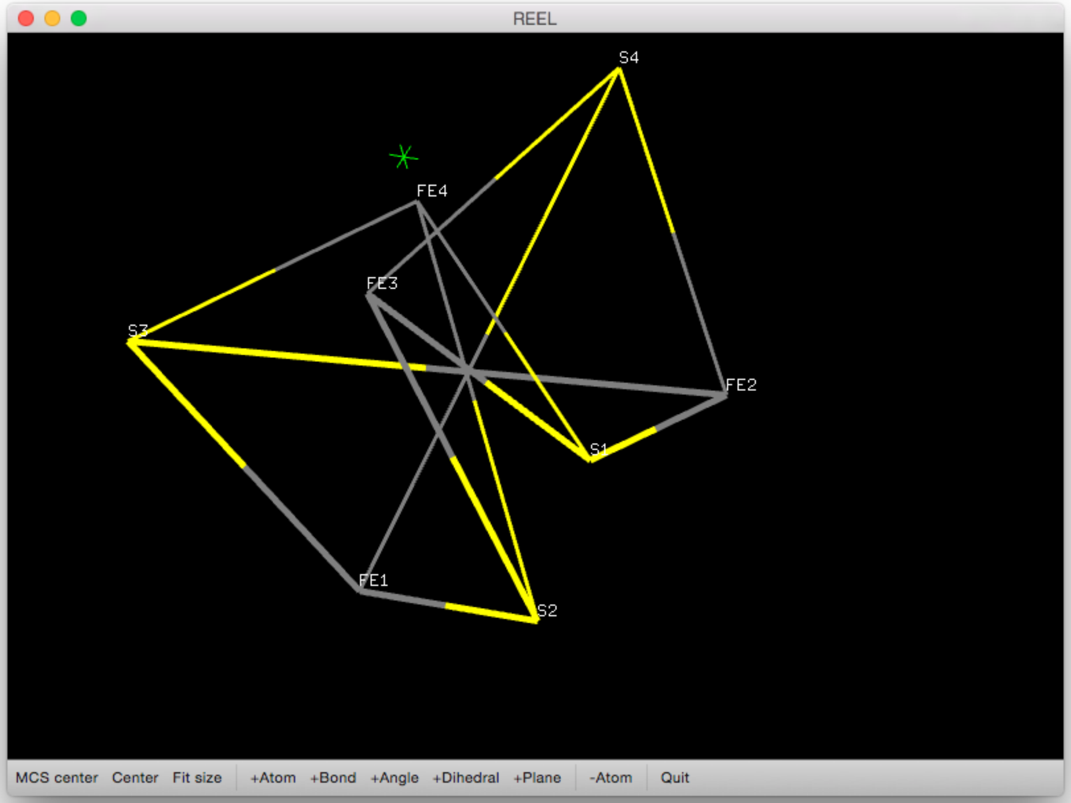

Figure S2 Loading coordinates from 2FLA into the standard Monomer Library restraints.

S2. Conquest search details

A conquest search that measures internal coordinate values requires a molecule specification with the geometry features listed in a QUEST query file and filter values. The query file and filter settings for the top of Table 1 are given in Figure S3 and Table S1, respectively.

```
T1  *CONN
NFRAG -99
AT1  S 3   T3                :XY  203  203
AT2  Fe 4   T4                :XY  203  263
AT3  S 3   T3                :XY  263  263
AT4  Fe 4   T4                :XY  263  203
AT5  S 3   T3                :XY  305  161
AT6  S 3   T3                :XY  161  305
AT7  Fe 4   T4                :XY  305  305
AT8  Fe 4   T4                :XY  161  161
AT9  S 1                    :XY  323  203
AT10  S 1                    :XY  143  263
AT11  S 1                    :XY  161  101
AT12  S 1                    :XY  305  365
BO  1  2  99
BO  1  4  99
BO  7  12  1
BO  8  5  99
BO  1  8  99
BO  3  4  99
BO  4  9  1
BO  4  5  99
BO  6  7  99
```

```
BO 2 3 99
BO 2 6 99
BO 2 10 1
BO 8 11 1
BO 3 7 99
BO 7 5 99
BO 8 6 99
GEOM
DEFINE SFeS1 3 2 1
DEFINE SFeS2 6 2 1
DEFINE SFeS3 1 8 6
DEFINE SFeS4 1 8 5
DEFINE SFeS5 1 4 3
DEFINE SFeS6 1 4 5
DEFINE SFeS7 6 2 3
DEFINE SFeS8 5 4 3
DEFINE SFeS9 6 7 3
DEFINE SFeS10 5 7 3
DEFINE SFeS11 5 8 6
DEFINE SFeS12 5 7 6
DEFINE SFe1 1 2
DEFINE SFe2 1 8
DEFINE SFe3 1 4
DEFINE SFe4 2 3
DEFINE SFe5 2 6
DEFINE SFe6 3 4
DEFINE SFe7 3 7
DEFINE SFe8 4 5
DEFINE SFe9 8 5
DEFINE SFe10 7 5
DEFINE SFe11 8 6
DEFINE SFe12 6 7
DEFINE FeSaa1 2 10
DEFINE FeSaa2 4 9
DEFINE FeSaa3 7 12
DEFINE FeSaa4 8 11
DEFINE SFea1 10 2 1
DEFINE SFea2 11 8 1
DEFINE SFea3 9 4 1
DEFINE SFea4 3 2 10
DEFINE SFea5 6 2 10
DEFINE SFea6 9 4 3
DEFINE SFea7 3 7 12
DEFINE SFea8 9 4 5
DEFINE SFea9 11 8 5
DEFINE SFea10 5 7 12
DEFINE SFea11 11 8 6
DEFINE SFea12 6 7 12
DEFINE FeSF1 8 1 2
DEFINE FeSF2 4 1 2
DEFINE FeSF3 4 1 8
DEFINE FeSF4 4 3 2
DEFINE FeSF5 7 3 2
DEFINE FeSF6 2 6 8
DEFINE FeSF7 7 6 2
DEFINE FeSF8 7 3 4
DEFINE FeSF9 4 5 8
DEFINE FeSF10 7 5 4
DEFINE FeSF11 7 5 8
DEFINE FeSF12 7 6 8
SYMCHK ON
ENANT NORMAL
END
```

Figure S3 QUEST query file for SF4 cluster linked via each Fe to one and only one sulphur atom.

Table S1 Table S1 Filter settings for the Conquest search

Filter	Value
3D coordinates determined	Yes

**R-factor**	0.05
**Only Non-disordered/Disordered**	Both
**No errors**	Yes
**Not polymeric**	No
**No ions**	No
**Only Single crystal structures/Powder structures**	Both
**Only Organics/Organometallic**	Both
